# Ought Women to Be on Hospital Committees?

**Published:** 1890-10-25

**Authors:** 


### Ought Women to be on Hospital Committees?

YES! By Miss Louisa Twining.

May I add one suggestion to the excellent article on "The Administrative Advance" in our hospitals, which all who have any knowledge of facts must know to be true?

The lack of interest in *all* matters of social public work is, alas! recognised and lamented in every department, not only in hospital management, but also in the duties of voting, electing, and finding the best people to carry out what are the responsibilities of all.

The suggestion is one that I have already often made, and which, I believe, will be carried out ere long. It is that *women* should be placed on the boards of management, for surely it needs no argument to prove that the duties you have assigned to these bodies are especially those which fall to the lot of woman in every household of the land, whether large or small. If men are too much occupied to come forward, women have the abundant leisure that is required, and who can be so fitted to work "in the cause of sickness and suffering as they?" I venture to think that the complaints as to diet, both of nurses and patients, would have been long since remedied had there been some women in authority to confer with the matron on these matters. Such details concerning household affairs, and, I may say, everything connected with the sick, is, and always has been, the province of woman, and we should cease to see empty chairs in the board-room on committee days, if a few well-qualified women were allowed to occupy them. My experience as a guardian of the poor has convinced me of this, and that these matters are, as a colleague once said to me, more fit for women than men to supervise.

At a recent meeting of a small country hospital, one member had the boldness to make this suggestion, which, naturally, caused some surprise, though the chairman of the meeting supported it by saying he had experience of the work which women could do in this way; but another influential member expressed his determination to retire from the committee were such a proposal carried out. As two ladies were present on this occasion (as governors or subscribers), this was hardly complimentary, or even courteous; but though the suggestion has failed for the present, no doubt is felt that it will ultimately be carried, to the certain advantage of both patients and staff.

I have named only, or chiefly, the domestic matters of housekeeping and nursing; but surely it can hardly be denied that the average intelligent woman has at least as much knowledge of sanitary and hygienic arrangements as the average man, not being medical.

I have heard of threats of "retiring from the board" being made by guardians if women were intruded upon them, but they have never been carried out, nor would they be, in this very similar case.

